# In Vivo Radionuclide Generators for Diagnostics and Therapy

**DOI:** 10.1155/2016/6148357

**Published:** 2016-12-12

**Authors:** Patricia E. Edem, Jesper Fonslet, Andreas Kjær, Matthias Herth, Gregory Severin

**Affiliations:** ^1^Department of Drug Design and Pharmacology, Faculty of Health and Medical Sciences, University of Copenhagen, Copenhagen, Denmark; ^2^Department of Clinical Physiology, Nuclear Medicine & PET, Rigshospitalet, Copenhagen, Denmark; ^3^Cluster for Molecular Imaging, Faculty of Health Science, University of Copenhagen, Copenhagen, Denmark; ^4^Center for Nuclear Technologies, Technical University of Denmark, Roskilde, Denmark; ^5^Department of Chemistry, Michigan State University, East Lansing, MI, USA; ^6^Facility for Rare Isotope Beams, Michigan State University, East Lansing, MI, USA

## Abstract

In vivo radionuclide generators make complex combinations of physical and chemical properties available for medical diagnostics and therapy. Perhaps the best-known in vivo generator is ^212^Pb/^212^Bi, which takes advantage of the extended half-life of ^212^Pb to execute a targeted delivery of the therapeutic short-lived *α*-emitter ^212^Bi. Often, as in the case of ^81^Rb/^81^Kr, chemical changes resulting from the transmutation of the parent are relied upon for diagnostic value. In other instances such as with extended alpha decay chains, chemical changes may lead to unwanted consequences. This article reviews some common and not-so-common in vivo generators with the purpose of understanding their value in medicine and medical research. This is currently relevant in light of a recent push for alpha emitters in targeted therapies, which often come with extended decay chains.

## 1. Introduction

The medical potential of radiation has been recognized nearly since the discovery of radioactivity. In the very first medical applications, isolated samples of naturally occurring *α*-emitters were used for the treatment of cancer (e.g., [1] or in review [2]). In these cases, the therapeutic benefits (or detriments) were partially due to the radioactive decay products, termed daughters, of the initially injected radionuclides, termed parents. By producing the daughter in the body, medically administered parents act as “in vivo generators.”

The term in vivo generator first appears in a conference abstract by Mausner et al. [3], discussing the use of targeted monoclonal antibodies (mAbs) with long-lived parent radionuclides that decay into short-lived daughter radionuclides. The concept was to combine the long half-life of the parent with the high decay energy of the daughter to achieve high-dose targeted radiotherapy. By introducing in vivo generators they proposed to overcome a physical limitation to radionuclide therapy where nuclides with high decay energies tend to have half-lives that are too short for targeted systemic therapies. Changes in biochemical interactions following decay are also exploited through the use of in vivo generators, exemplified by the ^81^Rb/^81m^Kr in vivo generator system. There the parent ^81^Rb^+^ (*t*_1/2_ = 4.3 h) accumulates in cells due to its chemical similarity to K^+^. Following a decay, the inert metastable daughter, ^81m^Kr (*t*_1/2_ = 13 s), leaves the cells via diffusion, allowing quantification of tissue perfusion [4].

These examples highlight the two main applications of in vivo generators, which are the same as the in vivo uses of radioactivity: diagnosis and therapy (briefly reviewed by Rösch and Knapp 2003 [5]). Overall, the advantage of in vivo generators is to combine nuclear and chemical properties of the parent and daughter nuclides to better diagnose or treat physiological conditions. This review is intended to cover many of the in vivo generators that have been used clinically and preclinically for imaging and therapy. The consequences and considerations of their use are also discussed, especially in light of the therapeutic *α*-emitters which have extended decay chains. Ideally highlighting the unique advantages afforded by in vivo generators will inspire future applications and expand the palette of nuclear and chemical properties available for medicine.

## 2. Release and Redistribution of Daughter Radionuclides

The most important consideration when employing an in vivo generator is the chemical consequence of the parent decay. In many cases, the chemical change from one atomic number to another is enough to cause a drastic difference between parent and daughter chemistry (e.g., a transition from alkali rubidium to noble krypton). However, even when the parent and daughter have nearly identical chemical behavior, as with transitions between lanthanides, there is still a possibility for a chemical change due to the atomic effects of nuclear decay. Molecular changes due to nuclear reactions are known as Szilard-Chalmers reactions, originally described by Szilard and Chalmers in 1934 as a means for separating isotopes of iodine after the chemical disruptions caused by neutron capture [6]. Since then there have been several Szilard-Chalmers type reactions described for the purpose of medical isotope production [7] and notably in generator reactions (e.g., [8]).

When extending the concept to in vivo generators, it is important to understand the circumstances that give rise to a difference between the parent and daughter chemistry. This is because, in most cases, medical radionuclides are used as labels on chemically specific “targeting” molecules such as peptides, antibodies, or receptor- and transporter-specific small molecules. When using an in vivo generator in these targeted systems, there are two opportunities for the daughter to separate from the parent, either as a result of the elemental differences between the parent and daughter or as a consequence of the physical and chemical disruption caused by the nuclear decay itself. Depending on the intended use of the generator, dislocation of the daughter from the targeting molecule can be an advantage or a disadvantage, and therefore it is important to understand when dislocation is expected and to understand the consequences of that dislocation.

In a series of papers, Zeevaart et al. described the physical considerations for when a chemical rearrangement is expected [9–12]. Their first approach was to look at whether or not the recoil of the daughter nucleus after *β*-decay, accounting for both the emitted neutrino and electron, could provide sufficient energy to dislodge the daughter from strong polydentate chelates. The general conclusion from their calculations is that in most instances the binding energy of the chelate is too high to be overcome by the small amount of energy involved in nuclear recoil. For example, for the pair ^90^Sr/^90^Y complexed to 1,4,7,10 tetraazacyclododecane 1,4,7,10 tetraacetic acid (DOTA), only 1% of all *β*-decays led to a dislocation of the daughter ^90^Y [10]. Internal transition (IT) decays are likewise consistent with stable chelation being retained under low energy recoil, for example, with the transition of   ^44m^Sc to ^44g^Sc leading to no observed dislocation of the daughter atom from stable chelate structures with DOTA [13]. For other decay modes, however, the physical recoil energy can far exceed the binding energy. This is the case with ^213^Bi *α*-decay where the recoil is on the order of 100 keV, compared to the binding energy of the daughter Pb(II) with the ubiquitous chelators DOTA or its amide derivative: 2-(4-isothiocyanotobenzyl)-1,4,7,10-tetraaza-1,4,7,10-tetra-(2-carbamonyl methyl)-cyclododecane (TCMC) of a few electron volts.

Electron capture (EC) decays are also efficient at releasing daughter nuclides from strong chelates, as observed with ^140^Nd and ^134^Ce. In these cases the Auger electron cascade following nuclear absorption of an inner shell electron causes atomic rearrangements that circumvent chelation. In similar Auger processes, such as the decay of ^165^Er, it is calculated that the autoionization process following transition to ^165^Ho leaves the daughter in an unstable and highly oxidized state, on average losing 7.6 electrons per decay [14]. Experimental observations of the EC decay of radiolanthanides are consistent with the conclusion that the daughters do not retain the parents' chemical state after the loss of so many electrons [8]. The same conclusion holds true for the similar process of internal conversion (IC). For example, in the decay of DOTA-bound ^166^Dy, 72% of all decays lead to an IC induced Auger cascade, and consistently 72% of daughter ^166^Ho atoms are found free from DOTA [10], showing that the delocalization is a Szilard-Chalmers reaction. As discussed by Zeevaart et al. [10] and Nath et al. [15], one possibility of counteracting the Auger-induced dislocation of the daughter atoms is to conjugate the metal ions via a ligand with an extensive delocalized *π*-electron system, such as heme. Such ligands are capable of quenching the so-called “Coulomb explosion” by transferring electrons to the metal center with comparable time scales to the Auger cascade.

Overall, the general rule to predict delocalization in in vivo generators is that, when the daughter and parent nuclide can both form stable complexes with the bioconjugate-chelate moiety, parent EC, IC, and alpha decays will all cause dislocation of the daughter, whereas *β* and IT decays tend to leave the daughter in the parent's chemical state.

## 3. Possible In Vivo Generators

Table 1 gives a list of some in vivo generators adapted from Rösch and Knapp [5]. Data were obtained from the National Nuclear Data Center [16].

## 4. In Vivo Generators for Diagnostics

### 4.1. General Principals

As stated above, the main purpose for applying in vivo generators for imaging and diagnostics is either to combine the long half-life of a parent nuclide with a short-lived daughter's diagnostic emission or to take advantage of a chemical change following the parent decay that can give information about the site of parent decay. The former is conceptually simple and is only complicated by the potential chemical change after decay. One great example where there is little to no concern over a chemical change after decay is with the use of ^44m^Sc, which decays via IT into its positron emitting daughter ^44g^Sc without changing its chemical state [13]. Thereby it becomes possible to consider ^44m^Sc as simply a long-lived positron emission tomography (PET) radionuclide. Despite the simplicity, this development relates to the critical arguments presented by Mausner et al.: there is an inverse relationship between the energy available for weak force mediated decays and nuclear lifetimes [3]. On the proton rich side of the chart of the nuclides, this fact along with the positron rest mass cutoff and the competitive decay mode of EC severely limits the number of long-lived *β* ^+^-emitters. This results in a shortage of long-lived PET radionuclides. To put it into perspective, the number of *β* ^+^-emitters with positron branching over 10% with half-lives between 12 h and 10 d is 10, whereas on the neutron rich side of the chart there are over 70 nuclides that undergo *β* ^−^ decay with the same half-life constraints [16]. Therefore, adding longer-lived in vivo generators of *β* ^+^-emitters makes a needed enhancement to the list of available nuclides for PET. Other PET in vivo generator pairs offer similarly long half-lives and chemistry as ^44m^Sc, namely, ^134^Ce and ^140^Nd, but these generators have the added trait that the daughter nuclides do not retain the parent chemical form but are freed after the initial EC decay [8]. Free radiometals can have misleading effects on PET and SPECT quantifications, as they often accumulate in disease sites and other tissues [17–20].

Not all of the diagnostic in vivo generators presented in Table 1 have been used in clinical or preclinical studies, but several have led to interesting results. Below are examples of some of the uses of diagnostic in vivo generators. The description starts with one of the first in vivo generators, ^81^Rb/^81m^Kr, that relies on the chemical change transitioning from rubidium to krypton to achieve the desired diagnostic information.

### 4.2. ^81^Rb/^81m^Kr

One of the first purposeful uses of an in vivo generator is ^81^Rb/^81m^Kr for imaging perfusion. Originally, the system was developed as an external generator of gaseous ^81m^Kr to be inhaled for pulmonary ventilation studies (e.g., [21, 22]). The 190 keV gamma from ^81m^Kr is well attuned for SPECT and gamma cameras, and the short half-life allows rapid repeat investigations of short physiological processes. One of the most important uses of ^81m^Kr, however, arises from the in vivo generator that results following injection of the parent ^81^Rb.

Rubidium mimics potassium and accumulates in cells, whereas krypton is inert, freely diffuses over cell membranes, and is carried away by blood flow. In 1971 Jones and Matthews [4] introduced the concept of measuring tissue perfusion with the in vivo generator by determining the rate at which ^81m^Kr is carried away from ^81^Rb containing tissues: the rate being proportional to perfusion. After a first pass through the lungs, the gaseous krypton leaves the blood stream. In practice, their method simply requires simultaneously quantifying ^81m^Kr and ^81^Rb in an organ of interest, which they show to be readily achieved with a sodium iodide or high purity germanium detector. They go on to show a proof-of-concept measurement of perfusion in a mock system where ^81^Rb is trapped on cation exchange resin, and ^81m^Kr is washed away by controlled flow of deionized water over the resin. Several other examples of the use of ^81^Rb/^81m^Kr as an in vivo generator appear in the literature [23–25].

### 4.3. ^52^Fe/^52m^Mn

The ^52^Fe/^52m^Mn in vivo generator potentially provides a wealth of biological information, although it is hindered by the fact that both the parent and the daughter emit positrons. As PET relies upon detection of annihilation photons at 511 keV, it is impossible for traditional PET to distinguish between ^52^Fe and ^52m^Mn decays. The respective positron branches are 55.5% for ^52^Fe and 96.8% for ^52m^Mn. Despite this difficulty, the pair remains desirable, or at least unavoidable, as a PET tracer for the distribution of biological iron. Iron transport in the body is particularly important as improper iron transport is linked to a number of diseases, and tracking the distribution of iron in supplements and remedies is important for drug development [26]. However, the only *β* ^+^-emitting isotope of iron with an appropriate half-life for biological tracing is ^52^Fe. In order to disentangle the contribution of ^52m^Mn to iron tracing by ^52^Fe, Lubberink and coworkers evaluated clinical data from injections of a sucrose-stabilized ^52^Fe along with blood sampling and determined tissue-dependent correction factors [27]. A similar approach was taken by Calonder et al. to investigate iron transport across the blood brain barrier in monkeys [28]. Those techniques were further adapted for a clinical evaluation of iron trafficking in patients with Wilson's disease, a condition related to improper metal ion transport [29].

### 4.4. ^140^Nd/Pr, ^134^Ce/La

^140^Nd and ^134^Ce have nearly identical properties: both are light lanthanides, have roughly 3 d half-lives, and decay purely by EC into short-lived *β* ^+^-emitting daughters. These nuclides are discussed for their therapeutic potential due to Auger emission from the parent decay followed by high-energy *β* emission from the daughters [30, 31]. The drawback of these pairs is that the parents' EC decay releases the daughters from targeting vectors. Dosimetrically, this is problematic because the high-energy positrons may be emitted away from targeted cells. Lubberink et al. assert that, in cases where targeting vectors are cellularly internalized, the daughter decays will occur within the targeted cell [31]. In preclinical animal models, one way to determine if the decays of the daughter occur in the same tissues as the parent is by PET imaging before and after sacrificing the animal. A premortem image will show the result of biological redistribution of the daughter, and the postmortem image will reveal the parent distribution. In cases where the daughter has high access to blood flow, a high level of redistribution would occur. When targeted tissues retain the daughters, this would indicate that the targeting vector would be appropriate for radiotherapy with in vivo generators. Contrastingly, when the targeted tissues do not retain the daughters, it may be appropriate to implement pretargeting (e.g., [32]) as the redistribution of the daughters could indicate that the targeting vector is accessible from the blood stream.

### 4.5. ^62^Zn/^62^Cu

Similar to ^52^Fe/^52m^Mn's role as a PET pair for tracing iron, the ^62^Zn/^62^Cu system is potentially useful as a tracer for biological Zn. Other uses include immunoPET [33] or small molecule imaging [34, 35]. In these cases, the positron branch in the parent is not as pronounced, only 8%. Therefore, PET imaging with the in vivo generator is dominated by positrons emitted by ^62^Cu, which has a 10-minute half-life in which to redistribute. More investigation into the consequences of redistribution would aid in the evaluation of ^62^Zn/^62^Cu as a PET radiolabel.

## 5. Therapeutic In Vivo Generators

### 5.1. General Principals

Targeted radiotherapy can be defined as the use of radiolabeled molecules as a means to deliver a cytotoxic amount of radiation to a disease site to treat a pathological condition (i.e., cancer, rheumatoid arthritis) [36]. When radiation interacts with biological tissues its energy is absorbed by the surrounding tissues and if sufficient, an orbital electron is ejected resulting in ionization. The ionized molecules (typically water) result in the formation of free radicals which can then cause damage to cellular components (e.g., DNA) resulting in cell death [37]. This process takes advantage of high-energy emissions such as *α*-particles, *β*-particles, or lower energy Auger electrons as a means to destroy cellular DNA.

Ultimately the goal of radiotherapy is to deliver a maximum radiation dose to the unhealthy cells while minimizing the radiation endured by healthy cells. One important factor that must be considered is the linear energy transfer (LET), which describes the ratio between the amount of energy transferred and the distance travelled by the radiation product. An advantage of *α*-emitters is that they possess a high LET when compared to *β*-emitters. For instances, *α*-particles can have LET values ranging from 80 to 100 keV/*µ*m while *β*-particles have an LET of 0.2 keV/*µ*m [38]. Given these constraints it is important to consider the targeting vector used when developing targeted radiotherapeutic agents. Criteria such as selectivity, specificity, and internalization need to be considered as well as the tumor size in the case of oncological disorders. The range of *α*-particle can be as little as a few cell diameters (40–80 *µ*m) making it possible to target small cell clusters, micrometastases, and single cells [38, 39]. On the other hand, *β*-particle emissions have a much longer path range (0.8–5 mm) and so the radiation is not necessarily deposited in the same location as the targeting vector, which can result in damage to the surrounding healthy tissue [38, 40].

Despite this, the majority of targeted radiotherapeutic agents used in the clinic are *β*-emitters [41]. In fact in 2013 the first *α*-emitting radioisotope that was approved for routine clinical use was ^223^Ra which has received approval from the Food and Drug Association (FDA) and the European Commission (EC) [42]. This discrepancy is likely due to the fact that most *α*-emitting radionuclides possess half-lives that are not suitable for clinical applications [36]. First the short half-lives limit the use targeting vectors that have long blood circulation times such as mAbs. Conversely isotopes with longer half-lives can be detrimental due to their high LET where there is a greater risk for toxicity as a result of irradiating healthy tissues during circulation. One solution is to perform direct injections into the diseased tissue; however, this is not always possible in the case of micrometastases. A way to circumvent this issue is to use in vivo generators.

An advantage of the in vivo generator system when applied to radiotherapy is that the longer-lived parent isotope can be cleared from blood circulation and healthy tissues while its high LET daughters will accumulate in the diseased tissue. This will result in a lower radiation dose to the healthy tissues and maximize the radiation dose at the target site. In particular, the amount of radioactivity administered can be minimized [39]. The challenges encountered during the use of in vivo generators for radiotherapy are similar to those encountered for diagnostic imaging. Achieving adequate specific activity; incorporating ligands that form stable complexes with both parent and daughter isotopes; and reducing toxicity due to isotope loss as a result of recoil energy during decay from parent to daughter are all issues that need to be carefully considered before applying this approach [43].

Below are some examples of in vivo generators that have been used for preclinical and clinical applications of radiotherapy. While each pair suffers from some of the challenges described above efforts have been made to overcome these issues or take advantage of them for a positive outcome.

### 5.2. ^212^Pb/^212^Bi

Lead-212 has been investigated as an in vivo generator for preclinical and clinical therapeutic applications using a variety of mAb systems for direct targeting and pretargeted radiotherapy [44–46]. Typically it has been used to target the human epithermal growth factor receptor (HER2) [45]. It undergoes *β* ^−^ decay producing ^212^Bi, *α*-emitter with a half-life of 60 min (Table 1). It is typically incorporated into biological molecules using chelating ligands such as DOTA and 2-(4-isothiocyanotobenzyl)-1,4,7,10-tetraaza-1,4,7,10-tetra-(2-carbamonyl methyl)-cyclododecane (TCMC) [47–49].

There have been difficulties in using this system due to instability of the ^212^Bi daughter. It has been reported from multiple studies that there is a 30–40% loss of ^212^Bi after 4 h due to the instability of the [^212^Bi]Bi-DOTA complex [44, 49]. The thermodynamic stability of the parent Pb-DOTA complex and the daughter Bi-DOTA complex was tested using the ^203^Pb(II) and ^206^Bi(III) isotopes. Interestingly rate of chemical exchange of these complexes was very slow aqueous solutions (pH 4-10) [48]. The calculated recoil energy of the ^212^Bi nucleus is not sufficient for breaking a chemical bond and so the instability of the complex is attributed to the poor kinetic stability of the ^212^Bi-DOTA complex [39]. This instability is caused by the radiative events that occur during isotopic decay, more specifically the internal conversion of the *γ*-rays emitted by the nuclide [48]. It has also been shown that direct labeling of DOTA conjugates with ^212^Bi is less efficient than other chelators such as diethylenetriaminepentaacetic acid (DTPA) in the presence of other complexing agents such as citric acid [50].

As a result any of the free ^212^Bi released will then be taken into the nontargeted healthy tissues such as the kidneys [39]. Attempts have been made in producing ligands that serve as stable chelators for both ^212^Pb and ^212^Bi. Ligands such as dioctyl terephthalate (DOTP) have shown reassociation with ^212^Bi, yet ^212^Pb showed decreases stability at concentrations below 1 mM; therefore, its use is only suitable for tracers not requiring high specific activity [39]. Nevertheless, ^212^Pb/^212^Bi in vivo generators have been shown to be more effective when applied to internalizing mAbs such as trastuzumab when compared to noninternalizing or nonspecific mAbs. One of the advantages of this system when compared to the *β* ^−^-emitting ^90^Y derivative is that the absorbed dose at the tumor site is higher, taking advantage of the higher LET of *α*-particles [45]. These promising preclinical results have inspired further clinical studies. [^212^Pb]Pb-TCMC-Trastuzumab has shown promise as a therapeutic agent over *β*-emitting isotopes following clinical evaluation [46].

### 5.3. ^225^Ac

Actinium-225 has shown promise as an in vivo generator system in preclinical and clinical applications. It is an isotope with a 10 d half-life that undergoes *α*-decay and produces three *α*-emitting radioisotopes: ^221^Fr (*t*_1/2_ = 4.9 min), ^217^At (*t*_1/2_ = 32.3 ms), and ^213^Bi (*t*_1/2_ = 46 min) [38]. Preclinical evaluation of ^225^Ac-DOTA-trastuzumab has shown increased survival rates in murine models of ovarian cancer [38]. Initially poor in vitro stability and low radiochemical yields were observed when the mAb was first functionalized with the DOTA ligand prior to radiolabeling. This was overcome by applying a two-step approach where the chelating ligand is first labeled with the metal and then it is conjugated to the mAb. Although high RCY yields (ca. 90%) are achieved in the first step, conjugation to the antibody is less efficient resulting in low apparent specific activity [38]. This can be problematic where high specific activity is required; however, in general ^225^Ac therapy is better suited for internalized mAbs since the ^221^Fr isotope, for example, is no longer able to be bound by the chelating ligand and diffuses away from the targeting vector [38]. In analysis of ^225^Ac labeled mAb, lintuzumab has shown that renal toxicity and an anemia could be a concern when using high doses in nonhuman primates [51]. Clinical trials using low doses of this agent are ongoing.

Another strategy to combat the diffusion of daughter isotopes is to employ encapsulating liposomes, nanoparticles to sequester the radioactivity at the target site. For example, pegylated phosphatidylcholine-cholesterol liposomes of different sizes and charges were used to entrap ^225^Ac. The ionic daughters produced following radioactive decay (Fr^1+^, At^1−^, and Bi^3+^) are not expected to diffuse from the liposome. The retention of the last produced daughter ^213^Bi was much better in the larger sized liposomes. The use of these liposomes is better suited for locoregional therapy [52]. Perhaps a more applicable strategy involves using multilayered encapsulating nanoparticle that is then conjugated to a mAb. In this strategy the nanoparticle conjugate is able to contain the decay daughters of ^225^Ac while binding to antibody targets in vivo [53]. An advantage of these multifunctional nanoparticles is their chemical properties for the containment, functionalization, and imaging [53].

### 5.4. ^227^Th

Thorium-227 has demonstrated its utility as an in vivo generator. One of its advantages is that it can be readily obtained from a ^227^Ac generator at low costs. It has a 18.7 d half-life and decays via *β*-particle emission forming numerous *α*- and *β*-emitting isotopes: ^223^Ra, ^219^Rn, ^215^Po, ^211^Pb, ^211^Bi, and ^207^Tl. One of its most clinically relevant daughter isotopes is ^223^Ra. This bone seeking alkaline earth metal has been clinically approved for radiotherapy due to its ability to accumulate in the bone [54]. Since it dissociates from ligand systems it cannot be used directly to label targeting vectors but use of the ^227^Th in vivo generator is a way around this. Similar to the other in vivo generators discussed thus far ^227^Th is best suited for internalizing targeting vectors because the ^223^Ra isotope dissociates from ligand systems. [^227^Th]Th-DOTA-trastuzumab has shown to be effective in inhibiting the growth of breast cancer xenografts in mice, yet it appears to be better suited for treating microscopic tumors as opposed to fast growing macrometastases [47, 55].

### 5.5. ^223^Ra

Because of the bone seeking properties of the alkaline earth metals Ra^2+^ has been used to treat bone metastases as it behaves similarly to Ca^2+^. One of the most recently clinically approved in vivo generators, used for treating bone metastases, has been the ^223^Ra agent Xofigo [56]. It is administered as solution of [^223^Ra]RaCl. The ^223^Ra isotope has a half-life of 11.4 d and decays via *α*-particle emission producing a number of *α* and *β* ^−^ emitting daughter isotopes (^219^Rn, ^215^Po, ^211^Pb, ^211^Bi, ^211^Po, and ^207^Tl) [54]. One of the challenges in using *β* ^−^-emitters for treating bone metastases is because their long radiation range damage to the bone marrow is always a risk; therefore, their use has been restricted to alleviating pain [54]. When compared to the *β* ^−^ emitting alkaline earth metal ^89^Sr^2+^, ^223^Ra^2+^ displayed much higher bone uptake in preclinical models [54]. The use of ^223^Ra is an improvement over tracers using ^224^Ra since the gaseous ^222^Rn daughter produced would diffuse away from the target site [54].

### 5.6. ^166^Dy/^166^Ho

The applicability of the ^166^Dy/^166^Ho in vivo generator system was first reported in 1994. Unlike the other in vivo generators used for radiotherapy discussed thus far the daughter isotope actually produces a *β* ^−^-particle that is useful for therapy. When the parent isotope ^166^Dy (*t*_1/2_ = 81.5 h) is coordinated to the DTPA ligand system high serum and in vivo stability is observed. This remains true of the complex following decay to the Ho (*t*_1/2_ = 26.6 h) daughter [57]. When complexed with ethylenediaminetetramethylene phosphonate (EDTMP) ^166^Dy has been shown to accumulate in skeletal tissue and therefore can be applied for marrow ablation [58].

## 6. Discussion and Conclusion

The development of in vivo generators as diagnostic and therapeutic radiopharmaceuticals has expanded the scope of radioisotopes used in nuclear medicine. Their use has served as a way to employ radioisotopes that would have otherwise been overlooked despite exhibiting desirable nuclear proprieties for radio imaging and therapy (e.g., emission type, emission energy, and LET in the case of therapy). One of the many advantages of these systems is that they can circumvent issues regarding short radiochemical half-lives by taking advantage of the longer-lived parent isotope. From a practical stand point this eliminates some of the time constraints that exist in preparing shorter-lived radiopharmaceuticals (i.e., ensuring that an on-site cyclotron is present, implementing short radiolabeling reactions, and limiting distances for transportation). From a biological stand point this allows for the use of slow clearing targeting vectors such as mAbs. Direct labeling of a slow clearing targeting vector with a short-lived therapeutic or diagnostic isotope would be futile as the radioisotope would decay before reaching its target site. Whereas incorporating the parent isotope, where possible, makes it possible to deliver the medically relevant short-lived isotope to the target following radioactive decay. As a result this method has resulted in the development of a number of mAb based radiopharmaceuticals incorporating various in vivo generator systems for preclinical and clinical use.

In addition to the challenges observed with short half-lives, less commonly used radioisotopes often have chemical proprieties that prevent them from being incorporated into targeting vectors through typical chelation strategies. This is evident in the case of ^223^Ra, which is typically used in its free form due to its inability to form stable complexes with commonly used chelators. By using the in vivo generator ^227^Th, the ^223^Ra daughter is produced at the target site following radioactive decay. One must take care when applying this approach, however, because this required the targeting vector to be internalized to prevent diffusion of the daughter isotope to other tissues.

This leads to one of the main challenges observed in developing in vivo generators for clinical use. The instability of the radiopharmaceutical that can result from the chemical transformation of one element to another is something that must be considered. Although this property has been leveraged as a benefit when applied to internalizing targeting vectors for therapeutic applications it can still be a detriment especially for diagnostic imaging. When the daughter isotope is released from the targeting vector it can diffuse into nontargeted tissues resulting in confounding imaging results.

With the recent approval of Xofigo the medical community is taking advantage of the use of *α*-emitters as a therapeutic tool. Continued efforts to test additional in vivo generators in clinical trials are ongoing. By taking advantage of the increased efficacy of *α*-emitting therapeutics in vivo generators have the advantage to improve therapeutic outcomes over their *β*-emitting counterparts in radioimmunotherapy, making this approach very promising for clinical oncology.

## Figures and Tables

**Table 1:** 

	Parent	Decay	*t*_1/2_	Daughter	Decay	*t*_1/2_	Use
Diagnostics	^44m^Sc	IT	60 h	^44^Sc	*β*+	4 h	PET
	^52^Fe	EC/*β*+	8.3 h	^52m^Mn	*β*+	21 min	PET
	^62^Zn	EC/*β*+	9.2 h	^62^Cu	*β*+	9.7 min	PET
	^81^Rb	EC	4.6 h	^81m^Kr	IT	13 s	SPECT
	^99^Mo	*β*−	66 h	^99m^Tc	IT	6.0 h	SPECT
	^134^Ce	EC	3.2 d	^134^La	*β*+	6.5 min	PET
	^140^Nd	EC	3.4 d	^140^Pr	*β*+	3.4 min	PET

Therapy	^66^Ni	*β*−	2.3 d	^66^Cu	*β*−	5.1 min	*β*
	^103^Pd	EC	16 d	^103m^Rh	EC	46 min	Auger
	^112^Pd	*β*−	21.04 h	^112^Ag	*β*−	3.14 h	*β*
	^166^Dy	*β*−	3.4 d	^166^Ho	*β*−	1.12 d	*β*
	^212^Pb	*β*−	10.64 h	^212^Bi	a	1.01 h	*α*
	^213^Bi	*α*	45.6 min	^209^Pb	*β*−	3.3 h	*α*
	^223^Ra	*α*	11.4 d	Chain			*α*
	^225^Ac	*α*	10.0 d	Chain			*α*
	^227^Th	*α*	18.7 d	Chain			*α*
